# Resection of pleural solitary fibrous tumors with distinct feeding vessels arising from the descending aorta: a case report

**DOI:** 10.1186/s13019-024-02872-y

**Published:** 2024-06-25

**Authors:** Yongsen Li, Zihao Lu, Wenxuan Hu, Yonghao Cao, Xin Lv, Jun Zhao, Chun Xu

**Affiliations:** Department of Thoracic Surgery, The First Affiliated Hospital of Soochow University, No. 899 Pinghai Road, Gusu District, Suzhou City, Jiangsu Province China

**Keywords:** Pleural solitary fibrous tumors, Feeding vessels, Case report, Angiography

## Abstract

**Background:**

Pleural solitary fibrous tumors (pSFTs) are rare mesenchymal pleural tumors with rich vascularity. Surgical resection is the cornerstone of pSFTs treatment, requiring careful preoperative imaging to delineate lesion extent and vascular supply including contrast-enhanced computed tomography and other examinations depending on its size and characteristics.

**Case presentation:**

The patient was a 34-year-old female with a mass measuring approximately 67 × 42 × 65 mm in the left posterior mediastinum. Intraoperatively, the mass demonstrated rich vascularity. Two veins originating from the abdominal cavity entered the lower pole, one converged from the superior pole, draining into the brachiocephalic vein. Additionally, two arteries arose directly from the descending aorta, while several veins drained into the intercostal veins. In response to unexpected intraoperative vascular findings, vascular clips and silk threads were used to ligate them. Subsequently, the tumor was successfully dissected, with approximately 600 ml of blood loss recorded during the 4-hour surgery. The patient exhibited a satisfactory postoperative recovery, and follow-up spanning over six months revealed no indications of recurrence or metastasis.

**Conclusions:**

We firstly present a case of successful resection of a pSFT in a 34-year-old woman with a distinct feeding vessel arising from the descending aorta and describe the related surgical procedures. This case highlights preoperative evaluation of mass vascularity based on contrast-enhanced computed tomography. When blood supply is challenging to clarify, angiography can offer additional details, especially for giant pSFTs. Despite this, thorough intraoperative exploration remains essential to detect unexpected vessels. Appropriate interventions should be customized based on the vascular origins and the surrounding anatomical structures.

## Background

Solitary fibrous tumors (SFTs) are rare spindle cell neoplasms with moderate biological potential, initially documented by Klemperer and Rabin in 1931 [1]. Derived from mesenchymal tissues and widely distributed in interstitial spaces, SFTs can manifest in various anatomical locations, including the retroperitoneum, mesentery, thoracoperitoneum, and gastrointestinal tract [2]. However, they are predominantly found in the pleura. Pleural solitary fibrous tumors (pSFTs) represent 4% of chest tumors, with no significant variations in incidence rates based on gender [3]. Typically, they present as asymptomatic masses characterized by flat oval or spindle-shaped cells lacking a distinct proliferation pattern, accompanied by dilated staghorn-type vasculature in the collagenous stroma. Currently, the standard treatment is surgical intervention. This report details a case of pSFTs in a 34-year-old woman with numerous feeding vessels, particularly originating from the descending aorta.

## Case presentation

A 34-year-old female patient from China was admitted after the identification of a mass in the left posterior mediastinum through routine chest computed tomography (CT) scans. The patient experienced shortness of breath after tensive activities without fever, cough, sputum, chest tightness, pain, nausea, vomiting, night sweats, or fatigue. Upon admission, the patient exhibited alertness, orientation, no significant chest tenderness, clear bilateral breath sounds, with reduced sounds on the left side. The patient did not show symptoms such as joint swelling or tenderness typically associated with Pierre-Marie-Bamberg syndrome.

**Fig. 1:**
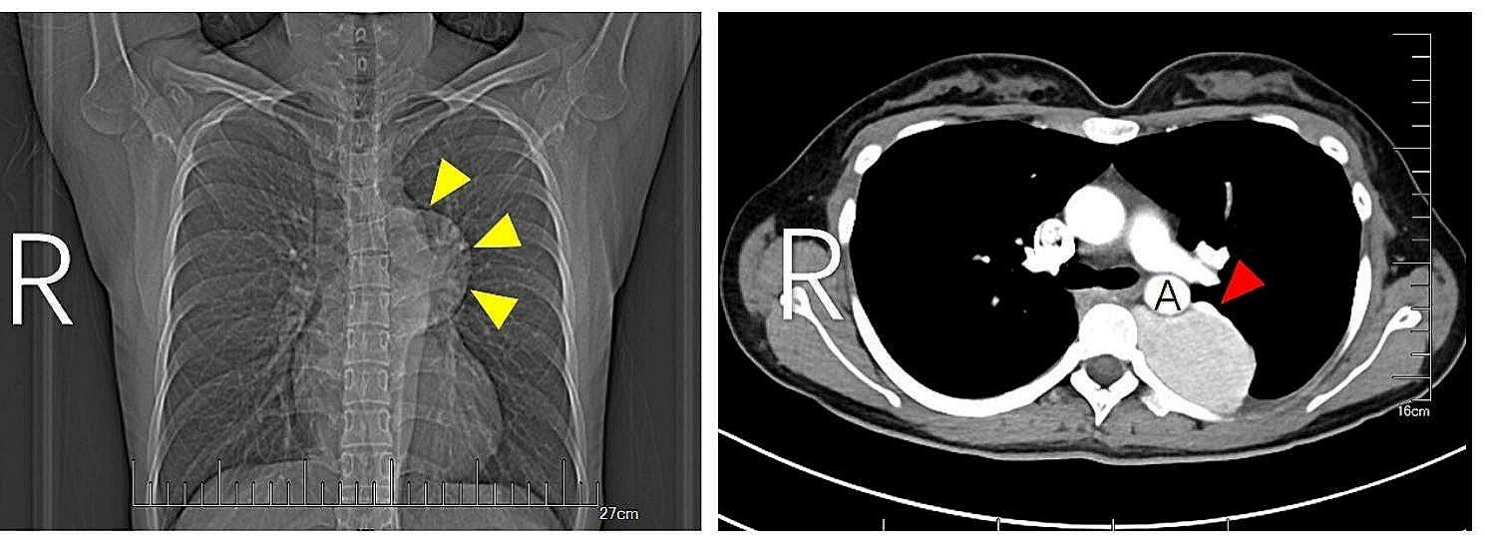
Chest radiography and contrast-enhanced computed tomography (CT) findings. Chest radiography demonstrated the presence of a mass within the left thoracic cavity, as indicated by yellow arrows. Contrast-enhanced CT scans exhibited a soft tissue density measuring approximately 39 mm×60 mm. A tortuous vein was observed encircling the surface of the mass, with drainage into the innominate vein. A, aorta

Thorough preoperative assessments were conducted, revealing no notable abnormalities in routine blood tests, which ruled out Doege-Potter syndrome. Pulmonary function tests showed obstructive ventilatory impairment at GOLD Stage 2, with a forced expiratory volume in one second (FEV1) of 1.92 L (70.8% predicted) and maximum voluntary ventilation (MVV) of 66.6% predicted. Contrast-enhanced CT scans demonstrated the presence of a tortuous vein enveloping the periphery of the mass and draining into the innominate vein, without any other feeding vessels identified (Fig. 1). The patient chose surgical intervention, refusing both vascular preoperative angiography and biopsy.

**Fig. 2:**
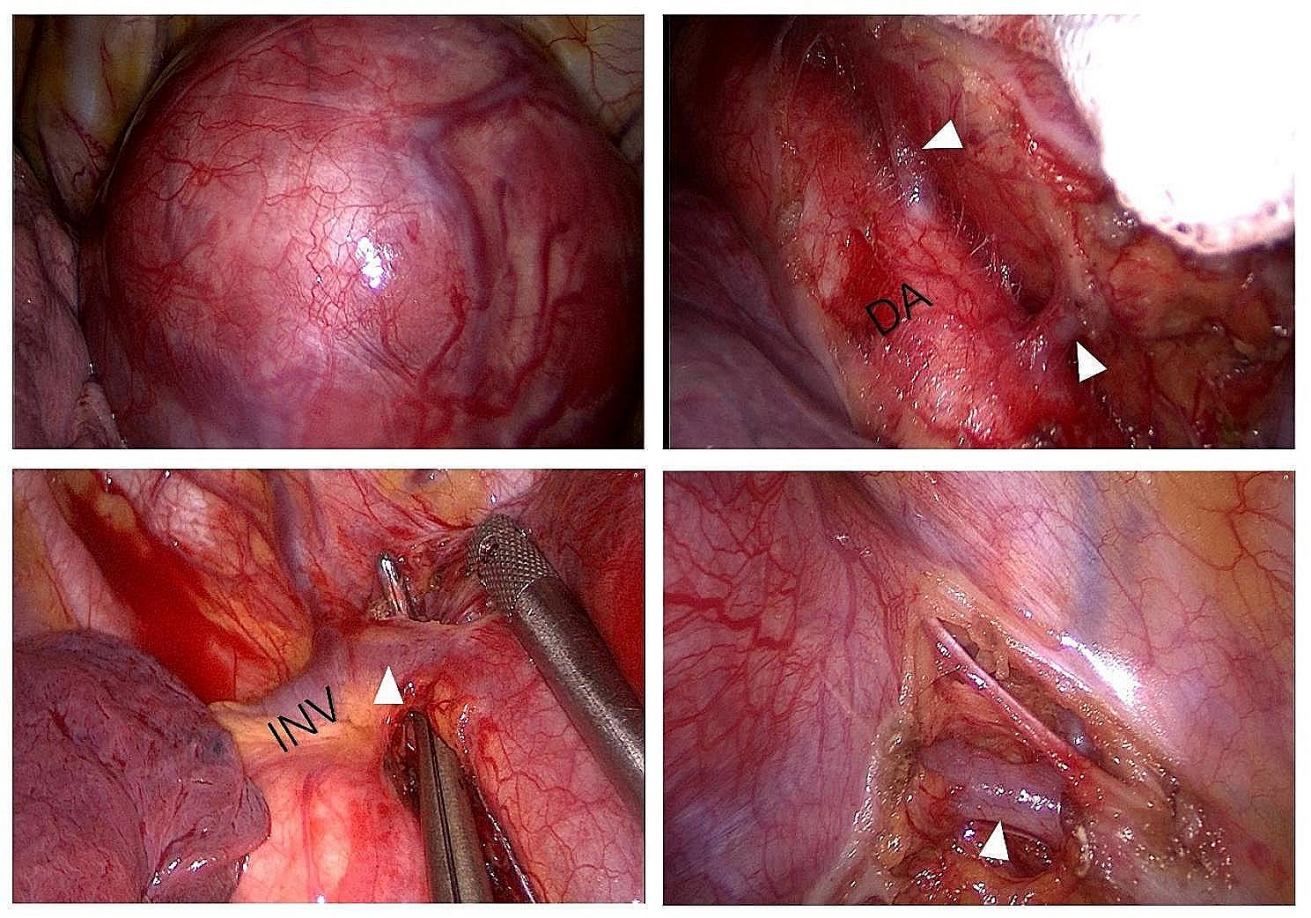
The intraoperative findings. Intraoperative observations revealed a tumor displaying abundant vascularity. Two arterial branches originating directly from the descending aorta were identified. The upper vein was observed draining into the innominate vein. Additionally, two venous tributaries from the abdominal cavity entered the lower pole of the mass. DA, descending aorta; INV, innominate vein

Thoracoscopic surgery was conducted through a 5 cm incision along the anterior axillary line at the fifth intercostal space. Intraoperatively, the mass was located in the left posterior thoracic cavity, situated below the spine and above the diaphragm. It exhibited partial cystic characteristics, was encapsulated, and closely associated with the descending aorta and posterior chest wall. To enhance the procedure, an additional 1.5 cm-incision was made at the seventh intercostal space along the posterior axillary line for thoracoscopy. Upon meticulous exploration, the mass exhibited rich vascularity (Fig. 2). Two veins from the abdominal cavity entered the lower pole of the mass, and the tortuous vein converged from the superior pole, draining into innominate vein. Additionally, two arteries originated directly from the descending aorta, and several veins drained into the intercostal veins. A controlled hypotensive strategy was initially employed when managing two short arterial branches directly from the descending aorta. Following silk ligature near the branches’ proximal end and reinforcement with vascular clips, transection was performed using an ultrasonic scalpel. Two inferior vessels were ligated at both ends before transection. Prior to transection with a linear cutting stapler, the upper vessel was dissected using angled forceps to create a gap. After adequate dissection within the fibrous tissue interstice using an ultrasonic scalpel, branches of intercostal vessels were transected using linear cutting staplers. Subsequently, the tumor was dissected along its margin. The wound was meticulously managed for hemostasis. Owing to the size and vascularity of the mass, approximately 600 ml of blood loss was recorded during the 4-hour surgery. The patient exhibited a satisfactory postoperative recovery, as demonstrated by X-ray revealing expanded lungs. Follow-up over six months revealed no signs of recurrence or metastasis.

**Fig. 3:**
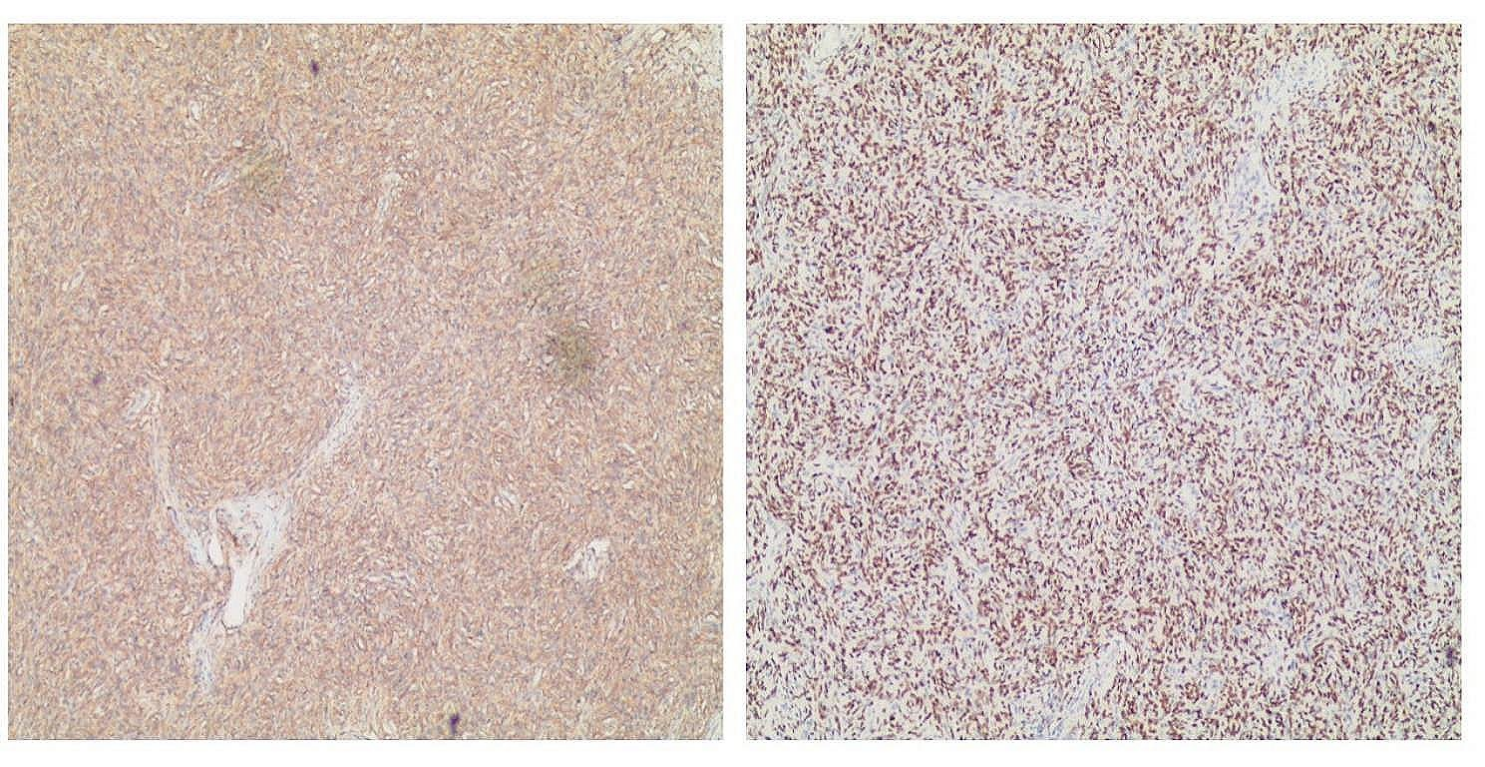
The histopathological findings. Immunohistochemical staining demonstrates the positive expression of CD34 and STAT6

Pathological examination confirmed an isolated fibrous tumor with a gray-white, tough, solid appearance. Immunohistochemical staining demonstrated positive expression of CD34, STAT6 (Fig. 3), and Catenin in tumor cells, while P63, S-100, and cytokeratin (CK) exhibited negative results. The Ki-67 index was approximately 5%. Besides, the pathological findings verified the successful removal of the tumor in this patient, with no remaining tumor at the surgical margins, thereby meeting the R0 criteria.

## Discussion and conclusions

Pleural solitary fibrous tumors (pSFTs) commonly manifest radiologically resembling other soft tissue tumors, displaying a uniform, lobulated, contrast-enhancing mass on cross-sectional imaging. In this case, contrast-enhanced CT imaging demonstrated that the tortuous vein encircles the surface of the mass, draining into the innominate vein. No other significant nutrient vessels were identified. There was a notable difference between the contrast-enhanced CT scan results and the intraoperative findings. This highlights the importance of carefully reviewing the preoperative contrast-enhanced CT scan results and conducting a thorough intraoperative exploration of the tumor’s vascular supply.

Preoperative angiography serves as an additional tool in the identification of major feeding vessels. Studies indicate that vascular pedicles from intercostal, internal mammary, or bronchial arteries may be found in up to 50% of SFTs [4]. Additionally, feeding vessels from the abdominal aorta [5] or celiac trunk [6] have been reported. The hypervascular nature of these masses can result in substantial hemorrhage, emphasizing the importance of preoperative evaluation of mass vascularity to facilitate bleeding-free resection. Certain authors recommend preoperative embolization of arterial vessels for giant masses, characterized by tumors with a diameter greater than 15 cm or when the tumor occupies over 40% of the hemithorax [7]. Preoperative embolization involves several techniques, such as micro-coils, polyvinyl alcohol granules or spongiosis granules and vascular plugs [4]. However, standardized indications for this procedure are not available.

In response to unexpected intraoperative vascular findings with thoracoscopy, we did not adopt the approach of angiography followed by embolization and subsequent surgery. Traditional methods of vascular clamping and silk ligature were utilized. A controlled hypotensive strategy was initially employed when managing short feeding vessels directly originating from the descending aorta. Following silk ligature near the vessel’s proximal end and reinforcement with vascular clips, transection was performed using an ultrasonic scalpel. The feeding vessels originating from the descending aorta have not been reported before, and their management is challenging, particularly due to frequent intraoperative bleeding of substantial volume, which impedes immediate intervention in minimally invasive surgeries. Upon reviewing the preoperative contrast-enhanced CT after surgery, we observed a vague and discontinuous punctate signal adjacent to the descending aorta, which indicated the potential presence of arteries branches. This situation prolongs the surgical duration and increases the risk of intraoperative bleeding. Preoperative angiography and embolization can significantly reduce the risk of bleeding during surgery, ensuring a smoother surgical procedure. While some scholars advocate preoperative angiography for giant SPF, additional angiography is recommended irrespective of tumor size if contrast-enhanced CT fails to assess vascular origin. Additionally, when encountering unexpected vessels during surgical procedures, it is crucial to thoroughly assess their risk of hemorrhage. Proper measures should be taken based on the vascular origin and the surrounding anatomical structures in manageable situations. While obtaining informed consent from patients is crucial during treatment, it should be tailored based on the patient’s conditions. To minimize significant risks to the patient, it is vital to strongly advocate for suitable examinations and treatment options.

This case introduces a novel finding regarding the feeding vessels originating from the descending aorta in pSFTs, along with the successful management during surgery. A comprehensive examination of contrast-enhanced CT images is essential to elucidate the vascularization of the tumor. In cases where the blood supply is not clearly visible, preoperative angiography may be warranted, especially for giant pSFTs, to mitigate the risk of intraoperative bleeding.

## Data Availability

No datasets were generated or analysed during the current study.

## Abbreviations

pSFTs: Pleural solitary fibrous tumors
CT: Computed tomography
SFTs: Solitary fibrous tumors
FEV1: Forced expiratory volume in one second
MVV: Maximum voluntary ventilation
CK: Cytokeratin
